# The Role of Lymphocyte Recovery Index in Prognosis Prediction for Locally Advanced Cervical Cancer With Radiation‐Induced Lymphopenia

**DOI:** 10.1002/cam4.70638

**Published:** 2025-02-14

**Authors:** Yi Li, Ao Liu, Xin Wang, Longxiang Guo, Yuanlin Li, Defeng Liu, Xiuli Liu, Zhichao Li, Minghuan Li

**Affiliations:** ^1^ Department of Radiation Oncology Shandong Cancer Hospital and Institute, Shandong First Medical University and Shandong Academy of Medical Sciences Jinan China; ^2^ Department of Radiation Oncology Qilu Hospital, Cheeloo College of Medicine, Shandong University Jinan China; ^3^ Department of Radiation Oncology Shandong Cancer Hospital, Cheeloo College of Medicine, Shandong University Jinan China; ^4^ Department of Oncology Dongying People's Hospital Dongying China; ^5^ School of Clinical Medicine Shandong Second Medical University Weifang China

**Keywords:** 18F‐FDG PET, cervical cancer, lymphocyte recovery index, radiation‐induced lymphopenia, spleen

## Abstract

**Background:**

In patients with locally advanced cervical cancer (LACC) undergoing concurrent chemoradiotherapy (CCRT), the high incidence of radiation‐induced lymphopenia significantly affects prognosis. There are significant variations in lymphocyte count (ALC) recovery patterns among patients, and their impact on prognosis remains unclear. This study aims to quantify the lymphocyte recovery patterns by the lymphocyte recovery index (LRI) and evaluate its prognostic value.

**Methods:**

This study reviewed patients with LACC who had ALCs available within 6 months post‐CCRT. Lymphopenia was graded using CTCAE 5.0, and lymphocyte recovery patterns were quantified using LRI (the ratio of ALCs at 6 months post‐treatment to baseline ALCs). Cox regression analysis was conducted to assess the correlation between LRI, other clinical factors, and survival. The dose–volume of bone marrow (BM) following pelvic radiotherapy was collected, and measurements of spleen standardized uptake value (SUV) and spleen‐to‐liver SUVmax ratio (SLR) were obtained from pre‐treatment 18F‐FDG PET/CT. Logistic regression analysis was used to identify independent risk factors for LRI.

**Results:**

A total of 180 patients were included retrospectively. During CCRT, 53 patients (29.4%) experienced G4 lymphopenia. The median LRI was 53.4% (range 13.2%–159.4%). Multivariable analysis revealed that LRI, G4 lymphopenia, and FIGO stage were associated with progression‐free survival (PFS) and overall survival (OS). Subgroup analysis revealed that the degree of lymphopenia (G4 and G1‐3) did not affect the correlation between LRI and PFS (P: 0.001 and 0.011) or OS (P: 0.003 and 0.043). Regarding FIGO stage, the impact of LRI on PFS (*p* < 0.001) and OS (*p* < 0.001) was primarily observed in patients with FIGO stage > II. Logistic analysis identified BM‐V10 > 96.0% and SLR > 0.90 as independent risk factors for LRI.

**Conclusion:**

In patients with LACC after CCRT, the LRI is associated with prognosis. Splenic metabolism and BM irradiation are associated with lymphocyte recovery.

## 1 Introduction

Cervical cancer is the fourth most common cause of cancer‐related deaths among women worldwide [1]. Currently, the primary treatment for patients with locally advanced cervical cancer (LACC) is definitive concurrent chemoradiotherapy (CCRT) [2, 3].

However, due to the high sensitivity of lymphocytes to radiation, radiation‐induced lymphopenia (RIL) often occurs after radiotherapy and affects survival in patients with various types of solid tumors, including cervical, pancreatic, and esophageal cancers. Compared with other types of solid tumors (pancreatic cancer: 34% [range 27%–45%], esophageal cancer: 35% [range 24%–45%]), patients with cervical cancer have a higher incidence of grade ≥ 3 RIL (58% [range 53%–61%]) [4, 5, 6]. In patients with cervical cancer undergoing definitive pelvic radiotherapy, the incidence of grade 4 (G4) RIL could even reach 33.1%, significantly impacting survival outcomes [7]. Although absolute lymphocyte counts (ALCs) typically gradually return to normal after treatment, patients with cervical cancer may take up to 5 years to recover to pre‐treatment levels [8].

Recent research has explored the correlation between lymphocyte recovery patterns from RIL and prognosis, but the conclusions have been inconsistent. In patients with pancreatic cancer, failure to recover from acute severe lymphopenia after CCRT was an independent predictor of poorer prognosis [9]. However, in patients with esophageal cancer, lymphocyte recovery after definitive chemoradiotherapy did not significantly impact prognosis [10]. This discrepancy may be attributed to differences in how radiotherapy affects lymphocyte recovery‐related organs depending on the tumor location. The spleen and bone marrow (BM), as crucial immune organs involved in lymphocyte regeneration and recovery, may have their baseline status and radiation dose affecting lymphocyte recovery patterns. PET/CT, as a functional imaging modality, can partially reflect the baseline status of the spleen [11].

In patients with cervical cancer, who have a higher incidence of RIL and longer recovery times, the relationship between lymphocyte recovery and prognosis remains unclear. Additionally, there are significant variations among patients in baseline ALCs and the recovery patterns of lymphocytes, which primarily include differences in the extent and rate of recovery among patients. It remains uncertain whether the baseline status and the radiation dose of immune‐related organs influence these factors. Therefore, it is essential to quantify lymphocyte recovery and further investigate the factors influencing it.

The aim of this study was to quantify the lymphocyte recovery patterns in patients with LACC after CCRT using the lymphocyte recovery index (LRI), evaluate its prognostic significance, and identify the factors associated with LRI.

## 2 Materials and Methods

### 2.1 Patients

**FIGURE 1:**
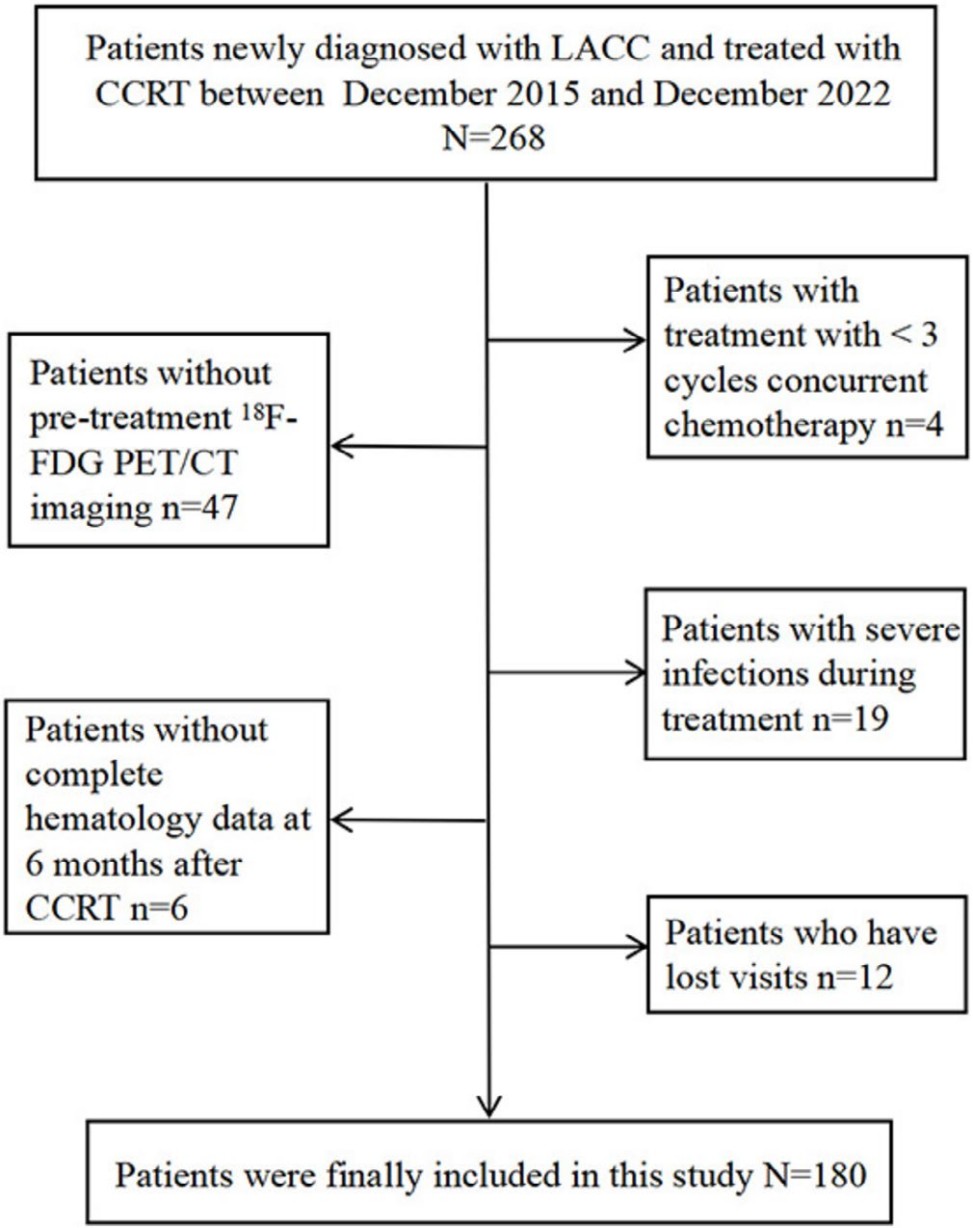
Recruitment and selection process of patients.

This retrospective study included 180 patients with LACC treated with CCRT between December 2015 and December 2022 at Shandong Cancer Hospital and Institute. Figure 1 shows the flowchart of the cohort selection process. The inclusion criteria were as follows: (1) age ≤ 75 years, (2) newly diagnosed and pathologically confirmed cervical cancer, (3) FIGO stage IB3‐IVA (2018 edition), (4) completion of a standard radiotherapy program, (5) treatment with 3–6 cycles of concurrent chemotherapy, (6) availability of hematology data within 6 ± 1 months after the completion of treatment, and (7) availability of 18F‐FDG PET/CT before CCRT. The exclusion criteria were as follows: (1) presence of other tumors; (2) presence of acute infection, autoimmune disease, or other significant comorbidities; and (3) failure to complete follow‐up or review as required.

### 2.2 Treatment and Follow‐Up

The treatment protocol adhered to both domestic and international guidelines and norms [12, 13]. External beam radiation therapy (EBRT) and brachytherapy (BT) were components of pelvic radiotherapy. EBRT was administered at a total dose of 45–50.4 Gy, delivered in fractions of 1.8–2.0 Gy each. The cumulative dose of EBRT and BT reached 80–90 Gy. Patients received chemotherapy, primarily weekly cisplatin (40 mg/m^2^), during EBRT. The follow‐up assessments were carried out roughly every 3 months during the first 2 years after therapy, followed by evaluations every 6 months for the subsequent 3–5 years, and then annually thereafter. The follow‐up primarily consisted of regular outpatient and inpatient evaluations, as well as telephone communication. In this study, progression‐free survival (PFS) was defined as the duration from the completion of CCRT to the initial diagnosis of disease progression, death, or the final follow‐up assessment. Overall survival (OS) was defined as the time until death from any cause.

### 2.3 Acquisition of Absolute Lymphocyte Counts

The retrospective review of patients' medical records was conducted to gather clinical information for further analysis. ALCs were obtained at three time periods: within 1 week before CCRT (baseline ALC), weekly during CCRT (weeks 1–8), and at 6 ± 1 months after CCRT (post‐treatment ALC). The LRI was calculated using the following formula:

$$\;\mathrm{LRI}=\frac{\text{post}-\text{treatment}\;\mathrm{ALC}}{\text{baseline}\;\mathrm{ALC}}\times 100\%$$

The lowest ALC value during CCRT was defined as the nadir. It was graded from G1 to G4 based on the lower normal limit of ALC in our hospital (1.1 × 10^3^ cells/μL) and the Common Terminology Criteria for Adverse Events (CTCAE) version 5.0. Specifically, 1100–800 cells/μL, 800–500 cells/μL, 500–200 cells/μL, and < 200 cells/μL correspond to G1, G2, G3, and G4 lymphopenia (ALC nadir), respectively.

### 2.4 PET/CT Scanning and Quantitative Analysis

Baseline 18F‐FDG PET/CT examinations were performed prior to CCRT. Participants fasted for at least 6 h to ensure normal blood glucose levels before receiving an intravenous injection of 18F‐FDG at 4.4 MBq/kg. After resting for 60 min, imaging was conducted using an integrated PET/CT system (Gemini TF Big Bore, Philips Healthcare). Whole‐body CT scans were acquired with dose modulation (150 mAs, 130 kV, matrix size: 512 × 512, slice thickness: 3 mm), followed by PET scans with a matrix size of 144 × 144 and a 1 min acquisition per bed position. Data were corrected for randoms, decay, and scatter before reconstruction. PET images were processed using attenuation correction and the iterative ordered subset expectation maximization method and were fused with CT images to generate multi‐plane whole‐body views.

In the pre‐treatment PET/CT examination, spheroid volumes of interest (VOIs) measuring 5 cm and 3 cm in diameter were respectively positioned at the center of the right lobe of the liver and the spleen. These VOIs were utilized to measure the maximum standardized uptake value (SUV_max_) and the mean standardized uptake value (SUV_mean_) in both the liver and spleen for each patient. The spleen‐to‐liver SUVmax ratio (SLR) was determined by dividing the SUVmax of the spleen by the SUVmax of the liver.

### 2.5 Acquisition of Dose–Volume Parameters

Extract dose parameters from the treatment planning system, primarily including the mean doses of BM (BM_mean_) and the volumes of BM receiving doses of 10, 20, 30, and 40 Gy (V10, V20, V30, and V40), determined by analyzing the dose–volume histogram.

### 2.6 Statistical analysis

The R package maxstat was utilized to determine the optimal cutoff value for LRI [14]. The calculated cutoff values were rounded off to the nearest integer. Kaplan–Meier (KM) analysis was used to calculate cumulative survival probabilities and to compare survival outcomes between different subgroups. Hazard ratios (HR) and 95% confidence intervals (CI) were calculated using univariable and multivariable Cox proportional hazard regression analyses. The factors associated with LRI were determined using univariable and multivariable logistic regression analyses. All statistical analysis was carried out using SPSS 27.0.1 and R software 4.3.2. The *p*‐value below 0.05 was considered statistically significant.

## 3 Results

### 3.1 Patient Characteristics

The study enrolled a total of 180 patients. The age of patients ranged from 22 to 75 years, with a median age of 53 years. The majority of patients (58.9%) exhibited an ECOG performance status score of 0. The median of the maximum tumor diameter (MTD) was 4.9 cm, with squamous cell carcinoma accounting for 162 cases (90.0%) and adenocarcinoma for 18 cases (10.0%). The majority of patients (69.4%) were in FIGO stage III–IV, with 99 patients (55.0%) having pelvic lymph node metastasis and 40 patients (22.2%) having para‐aortic lymph node metastasis. All patients underwent CCRT, with 25.6% receiving EBRT doses exceeding 50.4 Gy, and 48.9% receiving BT doses exceeding 40 Gy. The number of cycles of concurrent chemoradiotherapy ranged from 3 to 6 cycles; 99 patients (55.0%) received 3–4 cycles, and 81 patients (45.0%) received 5–6 cycles. The data mentioned above are presented in Table 1.

**TABLE 1:** Patient characteristics.

Characteristics	No. of patients (%)
Age, years	
≤ 53	93 (51.67)
> 53	87 (48.33)
ECOG	
0	106 (58.89)
1–2	74 (41.11)
FIGO stage	
≤ II	55 (30.56)
> II	125 (69.44)
MTD, cm	
≤ 4.9	94 (52.2)
> 4.9	86 (47.8)
Pathology	
SCC	162 (90.00)
ADC	18 (10.00)
Pelvic LN	
N0	81 (45.00)
N1	99 (55.00)
Para‐aortic LN	
N0	140 (77.78)
N1	40 (22.22)
BT dose, Gy	
≤ 40	92 (51.11)
> 40	88 (48.89)
EBRT dose, Gy	
≤ 50.4	134 (74.44)
> 50.4	46 (25.56)
Chemotherapy cycle	
≤ 4	99 (55.00)
> 4	81 (45.00)
Nadir‐ALC	
G1‐3	127 (70.56)
G4	53 (29.44)

Abbreviations: ADC, adenocarcinoma; ALC, absolute lymphocyte count; BT, brachytherapy; EBRT, external beam radiation therapy; ECOG, Eastern Cooperative Oncology Group; FIGO, Fédération Internationale de Gynécologie et d'Obstétrique; MTD, maximum tumor diameter; SCC, squamous cell carcinoma.

### 3.2 Lymphopenia During and After Treatment

**FIGURE 2:**
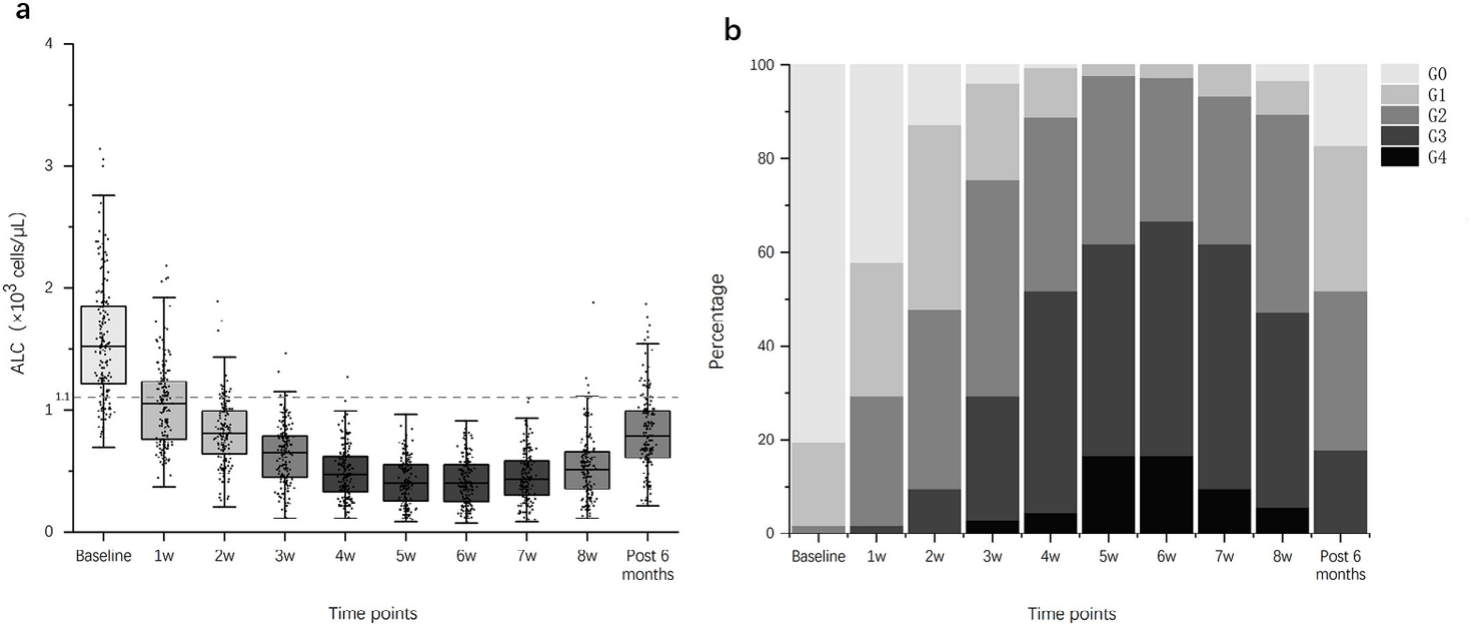
(a) The dynamic changes in absolute lymphocyte count (ALC) at baseline, during treatment (weeks 1–8), and at month 6 post‐treatment; (b) the percentage of patients at each time point with grade 0 (G0), G1, G2, G3, and G4 ALC nadir.

Following the initiation of treatment, the median ALCs gradually declined from baseline (1.52 × 10^3^ cells/μL), reaching a plateau by week 5. By week 8, there was no significant recovery observed, and even by month 6 after the end of treatment, the median ALCs (0.79 × 10^3^ cells/μL) had still not returned to normal levels (Figure 2a). The median ALCs were 1.05, 0.81, 0.65, 0.47, 0.40, 0.40, 0.43, and 0.51 (×10^3^ cells/μL) for weeks 1–8 during CCRT, respectively. During treatment, 53 patients (29.4%) experienced G4 ALC lymphopenia, whereas 127 patients (70.6%) experienced G1–3 ALC lymphopenia. By month 6 post‐treatment, 134 patients still had not recovered ALCs, including 56 patients (31.1%) with G1 lymphopenia, 61 patients (33.9%) with G2 lymphopenia, and 32 patients (17.8%) with G3 lymphopenia (Figure 2b).

### 3.3 Survival Outcomes by Lymphopenia and Lymphocyte Recovery

**FIGURE 3:**
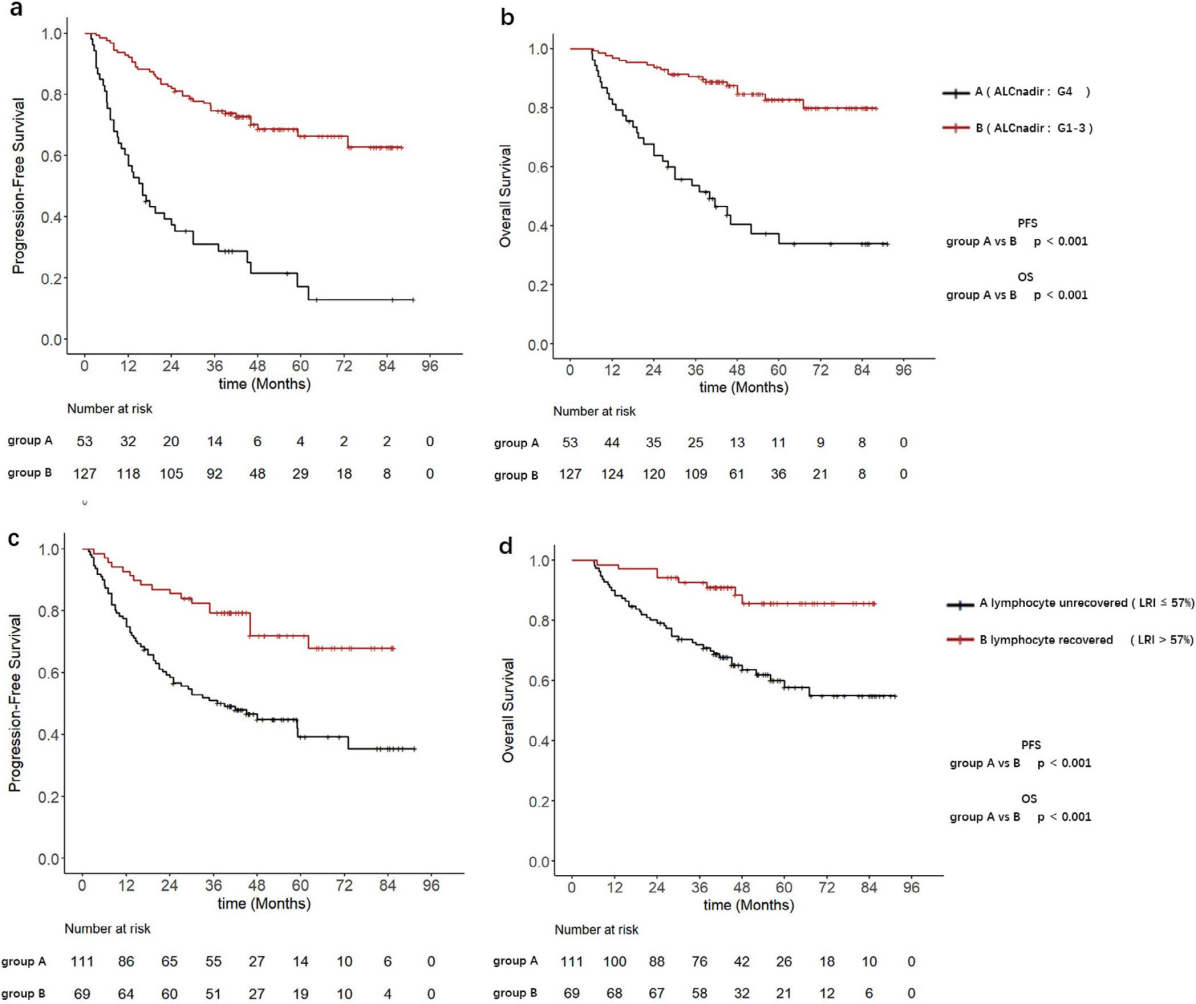
(a, b) Kaplan–Meier curves of different ALC nadirs group, for PFS and OS; (d, c) Kaplan–Meier curves of different LRI group, for PFS and OS.

Following the grading of lymphopenia during treatment, patients were divided into two groups based on ALC nadir: G4 lymphopenia (ALC nadir) and G1‐3 lymphopenia (ALC nadir). KM analysis revealed significant differences in PFS (*p* < 0.001) and OS (*p* < 0.001) between patients who experienced G4 lymphopenia during treatment and those who experienced G1‐3 lymphopenia (Figure 3a,b). Patients who experienced G4 lymphopenia exhibited lower 5‐years PFS rate (24.5% vs. 70.1%) and 5‐years OS rate (41.5% vs. 85.8%) compared with those who experienced G1‐3 lymphopenia.

The LRI was used to quantify the lymphocyte recovery patterns post‐treatment. The cutoff value of 57% for LRI was identified using the R package maxstat, based on the PFS (Figure S1). Patients were subsequently divided into two additional groups based on the LRI: inadequate lymphocyte recovery (LRI ≤ 57%) was considered as “unrecovered”, whereas adequate lymphocyte recovery (LRI > 57%) was considered as “recovered”. KM analysis demonstrated significant differences in PFS (*p* < 0.001) and OS (*p* < 0.001) between patients with unrecovered lymphocyte and those with recovered lymphocyte post‐treatment (Figure 3c,d). The 5‐years PFS rate (45.1% vs. 75.4%) and 5‐years OS rate (63.1% vs. 88.4%) were lower in patients with unrecovered lymphocyte compared with those with recovered lymphocyte.

To determine factors associated with PFS and OS, multivariable Cox analysis was performed to adjust for risk factors for all patients. The analysis revealed that FIGO stage > II, adenocarcinoma, G4 lymphopenia (ALC nadir) during treatment, and unrecovered lymphocyte (LRI ≤ 57%) post‐treatment independently predicted poorer PFS. Additionally, FIGO stage > II, MTD > 4.9 cm, G4 lymphopenia (ALC nadir), and unrecovered lymphocyte (LRI ≤ 57%) independently predicted poorer OS. It was observed that FIGO stage > II, G4 lymphopenia (ALC nadir), and unrecovered lymphocyte (LRI ≤ 57%) significantly influenced both PFS and OS. Subgroup analysis was then performed to explore the correlation between LRI and prognosis among patients with different ALC nadirs (G4 and G1‐3 lymphopenia) and FIGO stages ( > II and ≤ II) (Table 2).

**TABLE 2:** PFS and OS‐related univariable and multivariable analyses for all patients.

Variable	PFS				OS			
	Univariable analysis		Multivariable analysis		Univariable analysis		Multivariable analysis	
	HR (95% CI)	*p*	HR (95% CI)	*p*	HR (95% CI)	*p*	HR (95% CI)	*p*
Age ( > 53 vs. ≤ 53 years)	1.46 (0.94 ~ 2.27)	0.095			1.35 (0.77 ~ 2.36)	0.295		
FIGO stage ( > II vs. ≤ II)	5.80 (2.79 ~ 12.08)	**< 0.001**	4.93 (2.07 ~ 11.75)	**< 0.001**	6.17 (2.22 ~ 17.15)	**< 0.001**	4.17 (1.29 ~ 13.50)	**0.017**
ECOG (1–2 vs. 0)	1.33 (0.85 ~ 2.07)	0.207			1.28 (0.73 ~ 2.23)	0.389		
MTD ( > 4.9 vs. ≤ 4.9 cm)	2.00 (1.28 ~ 3.13)	**0.002**	1.20 (0.75 ~ 1.90)	0.450	3.15 (1.72 ~ 5.78)	**< 0.001**	2.13 (1.12 ~ 4.02)	**0.020**
Pathology (ADC vs. SCC)	2.20 (1.19 ~ 4.08)	**0.012**	2.48 (1.30 ~ 4.71)	**0.006**	1.77 (0.79 ~ 3.93)	0.162		
Pelvic LN (N1 vs. N0)	2.77 (1.70 ~ 4.51)	**< 0.001**	1.06 (0.57 ~ 1.96)	0.865	1.90 (1.05 ~ 3.41)	**0.032**	0.98 (0.50 ~ 1.90)	0.949
Para‐aortic LN (N1 vs. N0)	1.90 (1.18 ~ 3.08)	**0.009**	0.93 (0.54 ~ 1.59)	0.794	1.62 (0.87 ~ 3.01)	0.126		
BT dose ( > 40 vs. ≤ 40 Gy)	0.94 (0.61 ~ 1.46)	0.788			1.49 (0.85 ~ 2.61)	0.164		
EBRT dose ( > 50.4 vs. ≤ 50.4 Gy)	1.08 (0.65 ~ 1.78)	0.772			1.23 (0.66 ~ 2.29)	0.508		
Chemo cycles ( > 4 vs. ≤ 4)	1.54 (0.99 ~ 2.40)	0.057			1.83 (1.04 ~ 3.22)	**0.035**	1.32 (0.73 ~ 2.38)	0.361
Nadir‐ALC (G4 vs. G1‐3)	4.54 (2.91 ~ 7.09)	**< 0.001**	5.47 (3.37 ~ 8.88)	**< 0.001**	5.51 (3.11 ~ 9.79)	**< 0.001**	4.53 (2.49 ~ 8.24)	**< 0.001**
LRI ( ≤ 0.57 vs. > 0.57)	2.81 (1.66 ~ 4.75)	**< 0.001**	3.25 (1.89 ~ 5.59)	**< 0.001**	3.76 (1.77 ~ 8.02)	**< 0.001**	3.72 (1.73 ~ 8.01)	**< 0.001**

Abbreviations: ADC, adenocarcinoma; ALC, absolute lymphocyte count; BT, brachytherapy; EBRT, external beam radiation therapy; ECOG, Eastern Cooperative Oncology Group; FIGO, Fédération Internationale de Gynécologie et d'Obstétrique; LRI, lymphocyte recovery index; MTD, maximum tumor diameter; SCC, squamous cell carcinoma. Note: Parameters demonstrating statistical significance (*p* < 0.05) are highlighted in bold font.

### 3.4 Survival Outcomes by LRI in Patients with Different ALC Nadirs

**FIGURE 4:**
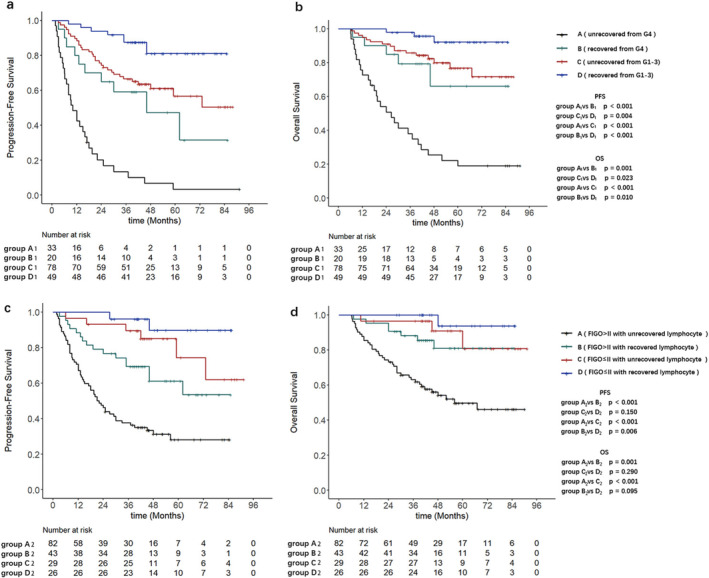
(a, b) The Kaplan–Meier curves of the 4 groups categorized by the LRI and ALC nadir, for PFS and OS; (d, c) the Kaplan–Meier curves of the additional 4 groups categorized by the LRI and FIGO stage, for PFS and OS.

Based on the LRI and ALC nadir, patients were divided into four groups: Group A1 (unrecovered from G4), Group B1 (recovered from G4), Group C1 (unrecovered from G1‐3), and Group D1 (recovered from G1‐3). KM analysis demonstrated significant differences in both PFS (*p* < 0.001) and OS (*p* = 0.001) between Groups A1 and B1. Compared with Group A1, Group B1 showed a lower 5‐years PFS rate (6.1% vs. 55.0%) and 5‐years OS rate (21.2% vs. 75.0%). Similarly, significant differences were observed between Group C1 and Group D1 in both PFS (*p* = 0.004) and OS (*p* = 0.023). Compared with Group D1, Group C1 showed significantly lower 5‐years PFS rate (61.5% vs. 83.7%) and 5‐years OS rate (80.8% vs. 93.9%) (Figure 4a,b).

Multivariable Cox analysis of the two subgroups demonstrated that unrecovered lymphocyte (LRI ≤ 57%), whether from G4 lymphopenia (ALC nadir) or G1‐3 lymphopenia (ALC nadir) at month 6 post‐treatment, were associated with PFS (HR 3.60 and 2.75) and OS (HR 4.24 and 3.61) (Tables 3 and 4).

**TABLE 3:** PFS and OS‐related univariable and multivariable analyses between Group A1 (unrecovered from G4 ALC nadir) and Group B1(recovered from G4 ALC nadir).

Variable	PFS				OS			
	Univariable analysis		Multivariable analysis		Univariable analysis		Multivariable analysis	
	HR (95% CI)	*p*	HR (95% CI)	*p*	HR (95% CI)	*p*	HR (95% CI)	*p*
Age ( > 53 vs. ≤ 53 years)	1.48 (0.77 ~ 2.82)	0.239			1.24 (0.59 ~ 2.59)	0.571		
FIGO stage ( > II vs. ≤ II)	6.63 (1.96 ~ 22.36)	**0.002**	6.51 (1.47 ~ 28.81)	**0.014**	4.81 (1.14 ~ 20.27)	**0.032**	2.78 (0.56 ~ 13.89)	0.212
ECOG (1–2 vs. 0)	1.15 (0.62 ~ 2.13)	0.663			1.16 (0.57 ~ 2.36)	0.676		
MTD ( > 4.9 vs. ≤ 4.9 cm)	1.51 (0.76 ~ 3.03)	0.240			2.18 (0.93 ~ 5.14)	0.073		
Pathology (ADC vs. SCC)	2.73 (1.11 ~ 6.72)	**0.029**	4.98 (1.82 ~ 13.62)	**0.002**	1.28 (0.45 ~ 3.69)	0.645		
Pelvic LN (N1 vs. N0)	2.65 (1.31 ~ 5.36)	**0.007**	1.09 (0.49 ~ 2.42)	0.825	2.48 (1.11 ~ 5.58)	**0.028**	1.81 (0.73 ~ 4.47)	0.199
Para‐aortic LN (N1 vs. N0)	1.34 (0.70 ~ 2.56)	0.375			0.94 (0.43 ~ 2.05)	0.882		
BT dose ( > 40 vs. ≤ 40 Gy)	1.10 (0.59 ~ 2.06)	0.753			2.05 (0.97 ~ 4.32)	0.059		
EBRT dose ( > 50.4 vs. ≤ 50.4 Gy)	1.06 (0.44 ~ 2.55)	0.890			1.66 (0.67 ~ 4.07)	0.272		
Chemo cycles ( > 4 vs. ≤ 4)	3.14 (1.62 ~ 6.06)	**< 0.001**	2.23 (1.12 ~ 4.44)	**0.022**	1.73 (0.85 ~ 3.51)	0.132		
LRI ( ≤ 0.57 vs. > 0.57)	3.61 (1.74 ~ 7.48)	**< 0.001**	3.60 (1.67 ~ 7.75)	**0.001**	4.30 (1.65 ~ 11.23)	**0.003**	4.24 (1.62 ~ 11.11)	**0.003**

Abbreviations: ADC, adenocarcinoma; BT, brachytherapy; EBRT, external beam radiation therapy; ECOG, Eastern Cooperative Oncology Group; FIGO, Fédération Internationale de Gynécologie et d'Obstétrique; LRI, lymphocyte recovery index; MTD, maximum tumor diameter; SCC, squamous cell carcinoma. Note: Parameters demonstrating statistical significance (*p* < 0.05) are highlighted in bold font.

**TABLE 4:** PFS and OS‐related univariable and multivariable analyses between Group C1 (unrecovered from G1‐3 ALC nadir) and Group D1 (recovered from G1‐3 ALC nadir).

Variable	PFS				OS			
	Univariable analysis		Multivariable analysis		Univariable analysis		Multivariable analysis	
	HR (95% CI)	*p*	HR (95% CI)	*p*	HR (95% CI)	*p*	HR (95% CI)	*p*
Age ( > 53 vs. ≤ 53 years)	1.05 (0.56 ~ 1.97)	0.884			0.87 (0.35 ~ 2.17)	0.766		
FIGO stage ( > II vs. ≤ II)	4.88 (1.91 ~ 12.51)	**< 0.001**	4.80 (1.52 ~ 15.16)	**0.007**	5.24 (1.21 ~ 22.69)	**0.027**	3.57 (0.81 ~ 15.79)	0.093
ECOG (1–2 vs. 0)	1.04 (0.54 ~ 2.01)	0.901			0.79 (0.30 ~ 2.07)	0.626		
MTD ( > 4.9 vs. ≤ 4.9 cm)	1.55 (0.83 ~ 2.91)	0.171			2.40 (0.96 ~ 5.98)	0.060		
Pathology (ADC vs. SCC)	2.21 (0.92 ~ 5.28)	0.075			2.34 (0.68 ~ 8.07)	0.177		
Pelvic LN (N1 vs. N0)	2.70 (1.36 ~ 5.35)	**0.004**	0.95 (0.41 ~ 2.19)	0.897	1.14 (0.46 ~ 2.81)	0.776		
Para‐aortic LN (N1 vs. N0)	1.82 (0.88 ~ 3.74)	0.104			1.93 (0.69 ~ 5.37)	0.210		
BT dose ( > 40 vs. ≤ 40 Gy)	0.67 (0.35 ~ 1.28)	0.223			0.88 (0.35 ~ 2.21)	0.792		
EBRT dose ( > 50.4 vs. ≤ 50.4)	1.68 (0.88 ~ 3.23)	0.116			1.97 (0.79 ~ 4.92)	0.145		
Chemo cycles ( > 4 vs. ≤ 4)	1.72 (0.90 ~ 3.27)	0.099			3.98 (1.41 ~ 11.26)	**0.009**	3.72 (1.32 ~ 10.50)	**0.013**
LRI ( ≤ 0.57 vs. > 0.57)	2.96 (1.36 ~ 6.44)	**0.006**	2.75 (1.25 ~ 6.01)	**0.011**	3.77 (1.10 ~ 12.94)	**0.035**	3.61 (1.04 ~ 12.53)	**0.043**

Abbreviations: ADC, adenocarcinoma; EBRT, external beam radiation therapy; ECOG, Eastern Cooperative Oncology Group; FIGO, Fédération Internationale de Gynécologie et d'Obstétrique; LRI, lymphocyte recovery index; MTD, maximum tumor diameter; SCC, squamous cell carcinoma; BT, brachytherapy. Note: Parameters demonstrating statistical significance (*p* < 0.05) are highlighted in bold font.

### 3.5 Survival Outcomes by LRI in Patients with Different FIGO Stages

Based on the LRI and FIGO stage, patients were further divided into four additional groups: Group A2 (FIGO>II with unrecovered lymphocyte), Group B2 (FIGO>II with recovered lymphocyte), Group C2 (FIGO≤II with unrecovered lymphocyte), and Group D2 (FIGO≤II with recovered lymphocyte). Subgroup analysis revealed that compared with Group B2, Group A2 exhibited poorer PFS (*p* < 0.001) and OS (*p* = 0.001). The 5‐years PFS rate was 31.7% for Group A2 and 65.1% for Group B2, whereas the 5‐years OS rates were 53.7% for Group A2 and 83.7% for Group B2. In contrast, there were no statistically significant differences in PFS (*p* =  0.150) and OS (*p* = 0.290) between Groups C2 and D2 (Figure 4c,d).

Multivariable Cox analysis of subgroups (A2 and B2) indicated that unrecovered lymphocyte (LRI ≤ 57%) independently predicted poorer PFS (HR 3.27%, 95% CI 1.85–5.78, *p* < 0.001) and OS (HR 4.21%, 95% CI 1.87–9.49, *p* < 0.001) (Table S1).

### 3.6 Parameters of Splenic 18F‐FDG Uptake and Bone Marrow Dose–Volume

The median SUV_mean_, SUV_max_, and SLR were 2.26 (range 1.49–3.43), 3.44 (range 2.20–6.35), and 0.90 (range 0.64–1.51), respectively. The median of BM_mean_ and BM (V10, V20, V30, and V40) were 28.5 Gy (range 10.6–38.9), 96.0% (range 49.3–97.8), 76.3% (range 34.2–87.8), 49.9% (range 23.3–64.1), and 25.7% (range 11.2–36.5), respectively (Table S2).

### 3.7 Predictors of LRI After CCRT

Using univariable logistic regression analyses, we found that SUV_mean_ > 2.26, SLR > 0.90, BM‐V10 > 96.0%, BM‐V20 > 76.3%, and BM‐V30 > 49.9% (all *p* < 0.05) were associated with unrecovered lymphocyte (LRI ≤ 57%). Subsequent multivariable analysis demonstrated that SLR > 0.90 (OR 2.55; 95% CI 1.30–5.02; *p* = 0.007) and BM‐V10 > 96.0% (OR 2.64; 95% CI 1.29–5.37; *p* = 0.008) independently predicted unrecovered lymphocyte (LRI ≤ 57%) (Table 5).

**TABLE 5:** Univariable and Multivariable Logistic analyses for lymphocyte recovery index (LRI).

Variable	Univariable analysis		Multivariable analysis	
	OR (95% CI)	*p*	OR (95% CI)	*p*
Age ( > 53 vs. ≤ 53 years)	1.14 (0.62 ~ 2.07)	0.679		
FIGO stage ( > II vs. ≤ II)	1.71 (0.90 ~ 3.26)	0.103		
ECOG (1–2 vs. 0)	1.14 (0.62 ~ 2.11)	0.670		
MTD ( > 4.9 vs. ≤ 4.9 cm)	1.60 (0.87 ~ 2.94)	0.128		
Pathology (ADC vs. SCC)	1.27 (0.45 ~ 3.56)	0.646		
Pelvic LN (N1 vs. N0)	1.60 (0.87 ~ 2.93)	0.128		
Para‐aortic LN (N1 vs. N0)	1.05 (0.51 ~ 2.16)	0.902		
Chemo cycles ( > 4 vs. ≤ 4)	0.83 (0.45 ~ 1.52)	0.548		
Nadir‐ALC (G4 vs. G1‐3)	1.04 (0.54 ~ 2.01)	0.915		
SUVmean ( > 2.26 vs. ≤ 2.26)	1.90 (1.03 ~ 3.50)	**0.040**	1.40 (0.71 ~ 2.74)	0.332
SUVmax ( > 3.44 vs. ≤ 3.44)	1.42 (0.78 ~ 2.60)	0.253		
SLR ( > 0.90 vs. ≤ 0.90)	3.05 (1.62 ~ 5.71)	**< 0.001**	2.55 (1.30 ~ 5.02)	**0.007**
BT dose ( > 40 vs. ≤ 40 Gy)	1.18 (0.64 ~ 2.15)	0.595		
EBRT dose ( > 50.4 vs. ≤ 50.4 Gy)	1.08 (0.54 ~ 2.16)	0.824		
BM‐V10 ( > 96.0 vs. ≤ 96.0%)	3.48 (1.84 ~ 6.59)	**< 0.001**	2.64 (1.29 ~ 5.37)	**0.008**
BM‐V20 ( > 76.3 vs. ≤ 76.3%)	2.04 (1.11 ~ 3.77)	**0.022**	1.53 (0.79 ~ 2.99)	0.208
BM‐V30 ( > 49.9 vs. ≤ 49.9%)	2.25 (1.22 ~ 4.17)	**0.010**	1.39 (0.69 ~ 2.80)	0.361
BM‐V40 ( > 25.7 vs. ≤ 25.7%)	1.15 (0.63 ~ 2.10)	0.646		
BM‐mean ( > 28.5 vs. ≤ 28.5 Gy)	1.01 (0.55 ~ 1.84)	0.971		

Abbreviations: ADC, adenocarcinoma; ALC, absolute lymphocyte count; BM, bone marrow; BT, brachytherapy; EBRT, external beam radiation therapy; ECOG, Eastern Cooperative Oncology Group; FIGO, Fédération Internationale de Gynécologie et d'Obstétrique; MTD, maximum tumor diameter; SCC, squamous cell carcinoma; SLR, spleen‐to‐Liver SUVmax ratio; SUV, standardized uptake value. Note: Parameters demonstrating statistical significance (*p* < 0.05) are highlighted in bold font.

## 4 Discussion

This study used the LRI to quantify lymphocyte recovery patterns in patients with LACC after CCRT and revealed that a lower LRI was associated with poorer survival outcomes. Patients undergoing CCRT commonly experience significant RIL, and their lymphocyte recovery patterns showed a prolonged time dependency. Moreover, the LRI was found to be associated not only with pelvic BM irradiation but also with baseline splenic metabolism. To the best of our knowledge, this is the first study to use the LRI to assess lymphocyte recovery in patients with LACC after CCRT and the first to demonstrate a connection between baseline splenic metabolism and lymphocyte recovery.

The systemic immunity plays a crucial role in cancer prevention and control. Lymphocytes are valuable markers for assessing the systemic immune response. Pathological evidence has confirmed that in patients with certain solid tumors, those with intense lymphocytic infiltration generally exhibit better PFS and OS compared with those without intense lymphocytic infiltration [15, 16, 17]. Therefore, RIL is increasingly garnering attention for its association with prognosis. Our previous research has substantiated that a low ALC nadir during treatment was significantly associated with poor PFS, OS and local recurrence‐free survival (LRFS) in patients with esophageal squamous cell carcinoma who underwent definitive radiotherapy [18]. Similar conclusions have been confirmed in other studies. In non‐small cell lung cancer, patients who experienced a lower ALC nadir after definitive radiotherapy showed significantly poorer event‐free survival and OS [19]. In patients with nasopharyngeal carcinoma who underwent CCRT, G4 lymphopenia has been identified as an independent prognostic factor for poorer distant metastasis‐free survival (DMFS) [20]. During pelvic radiotherapy, lymphocyte toxicity is more prevalent due to significant exposure of functional BM to the radiation field. Wu et al. [21]. found that patients with cervical cancer who underwent chemoradiotherapy commonly experienced severe and prolonged lymphopenia, and there was a correlation between lymphopenia and poorer PFS and OS. Verastegui et al. [8] demonstrated that patients with cervical cancer, compared with those with central nervous system tumors, experienced a more pronounced decline in ALCs following radiotherapy, with severe lymphopenia by week 5 of treatment. The recovery to pre‐treatment levels of ALCs took 60 months for T lymphocytes compared with 12 months for B cells. This is similar to our findings, where in this study, ALCs in patients with LACC who underwent CCRT reached its nadir by week 5 and had not yet returned to normal by month 6.

Considering the differences in baseline ALCs, post‐treatment lymphocyte recovery times, and recovery patterns among patients, we utilized the LRI (ratio of post‐treatment ALC at month 6 to baseline ALC) to quantify lymphocyte recovery, which also reflected the lymphocyte recovery capacity of patients. We found that an LRI ≤ 0.57 was significantly associated with both PFS and OS, regardless of whether patients experienced G4 or G1‐3 lymphopenia (ALC nadir). Specifically, in patients with FIGO stage > II, an LRI ≤ 0.57 was correlated with PFS and OS, whereas in patients with FIGO stage ≤ II, an LRI ≤ 0.57 did not show a significant correlation with prognosis. This confirmed the accuracy and specificity of the LRI in prognostic prediction and also reflected the poorer lymphocyte recovery capacity in patients with FIGO stage > II. Previously, Lee et al. [9]. found that in patients with advanced pancreatic cancer who underwent CCRT, failure to recover from acute severe lymphopenia within 6 months after treatment was significantly associated with poorer PFS and OS. They also observed a correlation between planning target volume and the recovery of ALCs. However, in research related to esophageal cancer, Deng et al. [10]. found that the recovery of ALCs at 6–8 weeks after chemoradiotherapy was not associated with OS. In contrast, Tseng et al. [14]. discovered that insufficient lymphocyte recovery by 6 months after CCRT was an independent predictor of poorer OS and PFS in patients with locally advanced esophageal cancer. Additionally, they found a correlation between BM‐V5 and lymphocyte recovery. In our study, we identified that the dose volume of BM including BM‐V10, BM‐V20, and BM‐V30, were correlated with LRI through univariable logistic regression analyses. After multivariable adjustment, a higher BM‐V10 ( > 96.0%) was an independent risk factor for a lower LRI ( ≤ 57%). Previously, Rose et al. [22] found that pelvic BM irradiation in patients with cervical cancer undergoing chemoradiotherapy correlated with increased hematologic toxicity (HT). Specifically, BM‐V10 > 95% correlated with a higher rate of grade 3 leukopenia. Additionally, Albuquerque et al. [23] found that if the V20 of the whole pelvis exceeded 80%, the risk of HT2+ increased by a factor of 4.5. Thus, higher volumes of pelvic bone marrow irradiation not only increase the incidence of hematologic toxicity but also impact subsequent recovery.

The spleen, as the primary site for the recruitment and activation of immune cells involved in inflammation, plays a crucial role in driving proinflammatory immune responses [24, 25]. Spleen FDG uptake can reflect cancer‐related inflammation and systemic immune status [26]. Elevated baseline uptake often indicates immune suppression, which may impact recovery from post‐treatment toxic effects. Therefore, we analyzed the correlation between LRI and splenic 18F‐FDG uptake. Univariable logistic regression analysis identified the parameters of splenic 18F‐FDG uptake associated with LRI, including SUV_mean_ and SLR. After multivariable adjustment, higher SLR was an independent risk factor for LRI. Currently, SLR has been shown to correlate with prognosis in patients with cervical cancer. De Jaeghere et al. [11] demonstrated that higher SLR was an independent predictor for predicting poorer disease‐free survival and pathological complete response. In the future, monitoring the splenic metabolism non‐invasively may provide new insights for predicting lymphocyte recovery and patient prognosis. Furthermore, considering the importance of lymph node evaluation in improving the prognosis of cervical cancer patients as reported in the previous studies [27, 28], we plan to monitor the metabolic levels of other immune organs, such as bone marrow and lymph nodes. This strategy is designed to uncover new prognostic and predictive markers, thereby facilitating the development of personalized treatment.

The clinical implications of this study lie in several key aspects. First, our findings underscore the prognostic value of the LRI in patients with LACC who develop RIL. Assessing post‐treatment lymphocyte recovery using LRI provides clinicians with valuable insights to guide subsequent treatment decisions. Moreover, our findings regarding the association of pelvic BM irradiation and baseline splenic metabolism with lymphocyte recovery emphasize the importance of designing treatment plans that minimize excessive pelvic BM irradiation, thereby reducing unnecessary immune system damage. In addition, non‐invasive monitoring of baseline splenic metabolism could be an effective approach for predicting the recovery of the immune system after treatment.

Our study has several limitations. First, as a single‐center retrospective study with a relatively small sample size, larger external validation or prospective studies are needed to confirm our findings. In future research efforts, we plan to collaborate with multiple centers to validate the prognostic role of LRI. Second, we did not investigate the changes in lymphocyte subtypes during treatment, which could be determined through flow cytometry.

## 5 Conclusions

In patients with LACC who underwent CCRT, LRI effectively quantified lymphocyte recovery patterns and served as a prognostic indicator. Higher volumes of bone marrow irradiation and increased splenic metabolism were associated with poorer lymphocyte recovery, resulting in prolonged recovery times for ALCs from RIL.

## Author Contributions

**Yi Li:** conceptualization (equal); data curation (equal); writing – original draft (equal). **Ao Liu:** conceptualization (equal); writing – original draft (equal). **Xin Wang:** conceptualization (equal); visualization (equal); writing – original draft (equal). **Longxiang Guo:** data curation (equal); formal analysis (equal); software (equal); visualization (equal). **Yuanlin Li:** data curation (equal). **Defeng Liu:** writing – original draft (equal). **Xiuli Liu:** data curation (equal); formal analysis (equal). **Zhichao Li:** visualization (equal); writing – original draft (equal). **Minghuan Li:** conceptualization (equal); writing – review and editing (equal).

## Ethics Statement

Our retrospective study abided by the rules of medical ethics, and the Institutional Review Board (IRB) of Shandong Cancer Hospital approved this study. The number for the ethical statement was SDTHEC2024006159. All patients were informed before treatment, agreed to receive concurrent CCRT and signed informed consent forms. We protected patient privacy and excluded patient identification information from our analyses.

## Conflicts of Interest

The authors declare no conflicts of interest.

## Supporting information


Figure S1.

Tables S1–S2.

## Data Availability

The data underlying this article cannot be shared publicly due to this study is based on the registry data from Shandong Cancer Hospital and Research Institute, which the authors do not own. The data will be shared on reasonable request to the corresponding author.
