# Supplementary figures and images for: The Role of Lymphocyte Recovery Index in Prognosis Prediction for Locally Advanced Cervical Cancer With Radiation‐Induced Lymphopenia
Source: Cancer Med. 2025 Feb 14;14(4):e70638. doi: 10.1002/cam4.70638 (PMC11827101; doi:10.1002/cam4.70638)

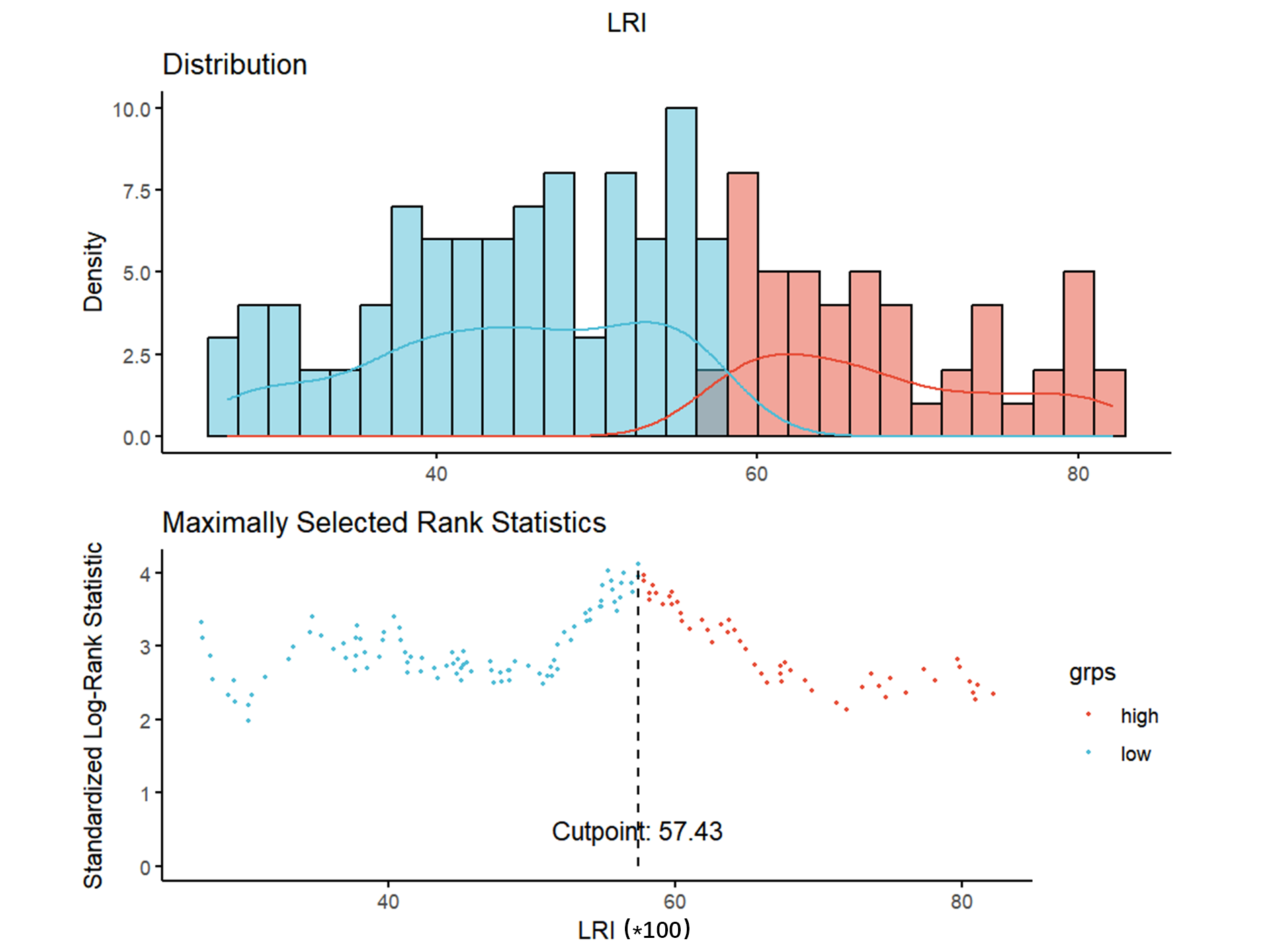

Supplement: Supplementary file 1 — Figure S1. [file CAM4-14-e70638-s002.tif]
