# Supplementary material for: The Role of Lymphocyte Recovery Index in Prognosis Prediction for Locally Advanced Cervical Cancer With Radiation‐Induced Lymphopenia
Source: Cancer Med. 2025 Feb 14;14(4):e70638. doi: 10.1002/cam4.70638 (PMC11827101; doi:10.1002/cam4.70638)
Supplement: Supplementary file 2 — Tables S1–S2. [file CAM4-14-e70638-s001.docx]

**Supplementary Table 1** PFS and OS-related univariable and multivariable analysis between A2 (FIGO stage >II with unrecovered lymphocyte) and B2 (FIGO stage >II with recovered lymphocyte)

| Variable | Progression-free survival | | | | Overall survival | | | |
| --- | --- | --- | --- | --- | --- | --- | --- | --- |
|  | Univariable analysis | | Multivariable analysis | | Univariable analysis | | Multivariable analysis | |
|  | HR（95%CI） | P | HR（95%CI） | P | HR（95%CI） | P | HR（95%CI） | P |
| Age (>53 vs. ≤53 years) | 1.36 (0.86 ~ 2.17) | 0.191 |  |  | 1.27 (0.71 ~ 2.28) | 0.412 |  |  |
| ECOG (1-2 vs. 0) | 1.15 (0.72 ~ 1.83) | 0.565 |  |  | 1.13 (0.63 ~ 2.02) | 0.674 |  |  |
| MTD (>4.9 vs. ≤4.9 cm) | 1.52 (0.95 ~ 2.44) | 0.082 |  |  | 3.16 (1.63 ~ 6.13) | **<0.001** | 2.62 (1.32 ~ 5.20) | **0.006** |
| Pathology (ADC vs. SCC) | 1.87 (0.98 ~ 3.57) | 0.056 |  |  | 1.35 (0.57 ~ 3.19) | 0.493 |  |  |
| Pelvic LN (N1 vs. N0) | 1.08 (0.61 ~ 1.91) | 0.783 |  |  | 0.72 (0.38 ~ 1.37) | 0.319 |  |  |
| Para-aortic LN (N1 vs. N0) | 1.15 (0.71 ~ 1.88) | 0.571 |  |  | 1.02 (0.54 ~ 1.91) | 0.952 |  |  |
| BT dose (>40 vs. ≤40 Gy) | 0.99 (0.62 ~ 1.57) | 0.967 |  |  | 1.57 (0.87 ~ 2.82) | 0.132 |  |  |
| EBRT dose (>50.4 vs. ≤50.4 Gy) | 1.02 (0.61 ~ 1.71) | 0.942 |  |  | 1.13 (0.59 ~ 2.14) | 0.715 |  |  |
| Chemo cycles (>4 vs. ≤4) | 1.36 (0.85 ~ 2.17) | 0.194 |  |  | 1.43 (0.80 ~ 2.58) | 0.227 |  |  |
| Nadir-ALC (G4 vs. G1-3) | 4.19 (2.61 ~ 6.74) | **<0.001** | 5.11 (3.13 ~ 8.35) | **<0.001** | 4.75 (2.60 ~ 8.68) | **<0.001** | 4.40 (2.38 ~ 8.15) | **<0.001** |
| LRI (≤0.57 vs. >0.57) | 2.54 (1.45 ~ 4.44) | **0.001** | 3.27 (1.85 ~ 5.78) | **<0.001** | 3.43 (1.53 ~ 7.67) | **0.003** | 4.21 (1.87 ~ 9.49) | **<0.001** |

Abbreviations: ECOG, Eastern Cooperative Oncology Group; MTD, maximum tumor diameter; ADC, adenocarcinoma; SCC, squamous cell carcinoma; BT, brachytherapy; EBRT, external beam radiation therapy; ALC, absolute lymphocyte count; LRI, lymphocyte recovery index

**Supplementary Table 2** PET/CT and Dose‑volume Parameters

| Characteristics | Median (Range) |
| --- | --- |
| PET/CT parameters |  |
| SUVmean | 2.26 (1.49 ~ 3.43) |
| SUVmax | 3.44 (2.20 ~ 6.35) |
| SLR | 0.90 (0.64 ~ 1.51) |
| Bone Marrow, % |  |
| BM-V10 | 96.0 (49.3 ~ 97.8) |
| BM -V20 | 76.3 (34.2 ~ 87.8) |
| BM -V30 | 49.9 (23.3 ~ 64.1) |
| BM -V40 | 25.7 (11.2 ~ 36.5) |
| BM -mean, Gy | 28.5 (10.6 ~ 38.9) |

Abbreviations: FDG-PET, fluorodeoxyglucose-positron emission tomography; SUVmax, maximum standardized uptake value; SUVmean, mean standardized uptake value; SLR, spleen-to-Liver SUVmax ratio; BM, bone marrow
